# The Autologous Hair Follicle and Its Secretome: A Multipotent Source for Cell-Based and Cell-Free Regenerative Therapies

**DOI:** 10.3390/ijms27104183

**Published:** 2026-05-08

**Authors:** Muneera Fayyad, Amatullah Fatehi, Sharon Samuel, Duhyun Han, Arpita Sathyanarayanan, Kendal Christie, Nazish Ahmed, Ian M. Rogers, Drew W. Taylor

**Affiliations:** 1 Acorn Biolabs Inc., Toronto, ON M5G 2N2, Canada; amatullah@acorn.me (A.F.); sharon@acorn.me (S.S.); christopher@acorn.me (D.H.); arpita@acorn.me (A.S.); kendal@acorn.me (K.C.); nazish@acorn.me (N.A.); 2 BMS Biomanufacturing Solutions Inc., Toronto, ON M5G 1N8, Canada; 3 Department of Physiology, University of Toronto, Toronto, ON M5S 3G9, Canada; rogers@lunenfeld.ca; 4 Lunenfeld-Tanenbaum Research Institute, Mount Sinai Hospital, Toronto, ON M5G 1X5, Canada

**Keywords:** autologous, regenerative medicine, hair follicle, stem cells, secretome, exosomes

## Abstract

Hair follicles (HFs) are highly accessible mini-organs that house diverse somatic and stem cell populations with broad therapeutic potential. In this study, we investigate the untapped utility of plucked HFs as a non-invasive tissue source for regenerative medicine. We demonstrate the successful isolation and expansion of keratinocytes and mesenchymal stem cells (MSCs) from plucked follicles using an enzyme-free explant culture method. HF-derived keratinocytes retained their epithelial identity and were efficiently reprogrammed into induced pluripotent stem cells (iPSCs). These iPSCs were further directed toward definitive endoderm and pancreatic progenitor fates, confirming their robust autologous regenerative capacity. Flow cytometric analysis of HF-MSCs validated a characteristic mesenchymal profile, and these cells exhibited classical trilineage plasticity alongside the ability to differentiate into dopaminergic neural progenitors. Furthermore, proteomic and vesicular characterization of the autologous HF secretome (aHFS) revealed a rich enrichment of regenerative cytokines and exosomes. The aHFS demonstrated potent wound-healing bioactivity in vitro. Collectively, these findings establish the plucked hair follicle as a highly practical, scalable source for both cell-based and cell-free therapies, highlighting the clinical value of early-stage follicular biobanking for personalized medicine.

## 1. Introduction

Hair follicles (HFs) are highly specialized, dynamic mini-organs characterized by life-long cyclic self-renewal. Their distinct microanatomical niches harbor diverse stem cell populations that drive transitions through phases of growth (anagen), regression (catagen), and rest (telogen) [1]. During the anagen phase, bulge stem cells migrate to the follicular bulb [1,2,3], differentiating into outer root sheath (ORS) and inner root sheath (IRS) cells [4,5].

Following matrix cell exhaustion, the lower cycling region undergoes apoptosis, eventually leading to a quiescent telogen state before a new cycle initiates [1,3]. This intrinsic regenerative capacity positions the HF as an accessible and powerful model for clinical tissue repair [6,7,8,9].

The regenerative potential of the HF relies on a complex interplay between somatic cells and the multiple stem cell compartments. Keratinocytes populating the ORS maintain a progenitor-like state and actively participate in epithelial repair [10]. These cells also demonstrate remarkably high reprogramming efficiencies for generating induced pluripotent stem cells (iPSCs) due to their endogenous expression of epithelial transcription factors [11,12]. Concurrently, the dermal papilla houses mesenchymal stem cell (MSC)-like populations exhibiting classical multilineage plasticity and potent immunomodulatory effects [13,14]. The follicular bulge also acts as a niche for neural crest-derived stem cells (NCSCs) capable of broad ectodermal and mesodermal differentiation [15], alongside pluripotent-like (HAP) cells isolated from the upper ORS [7,16].

The therapeutic utility of HF-derived cells extends beyond direct tissue integration, relying heavily on paracrine mechanisms mediated by the cellular secretome [17,18]. This localized mixture of growth factors, cytokines, and extracellular vesicles is naturally adapted to the cutaneous environment [19]. Preclinical evidence indicates that secretomes from HF compartments, particularly their exosomal fractions, can stimulate angiogenesis, promote re-epithelialization, and enhance wound closure in dermal injury models [14,20]. Translating these cell-free systems into clinical applications offers a standardized approach that mitigates the safety risks associated with whole-cell therapies [21,22].

Despite the robust regenerative profile of HF derivatives, both intrinsic and extrinsic aging heavily compromise therapeutic efficacy. Extended in vitro expansion induces replicative senescence, triggering DNA damage and secretory skew that significantly diminish paracrine function and tissue repair capacity [23,24]. While some primary tissue secretomes exhibit resilience to chronological age [25], cumulative oxidative stress and cellular aging inevitably degrade the differentiation potential of adult stem cells [26,27]. Consequently, securing unpassaged, youthful cells through early-stage cryopreservation is critical to circumvent replicative decay and maximize the biological fidelity of autologous cellular therapies [28].

In this study, we investigate the regenerative capabilities of human hair follicles, focusing on both cellular and cell-free constituents. We present original experimental data detailing the isolation, expansion, and directed multilineage differentiation of HF-derived keratinocytes and MSCs, alongside their efficient reprogramming into iPSCs. We also characterize the complex protein and vesicular profile of the autologous hair follicle secretome (aHFS) and assess its functional capacity to accelerate wound healing in vitro. By integrating these findings, we aim to validate the hair follicle as an optimal, accessible cell source for personalized regenerative medicine and emphasize the utility of follicular biobanking.

## 2. Results

### 2.1. Keratinocyte Isolation and Characterization

**Figure 1:**
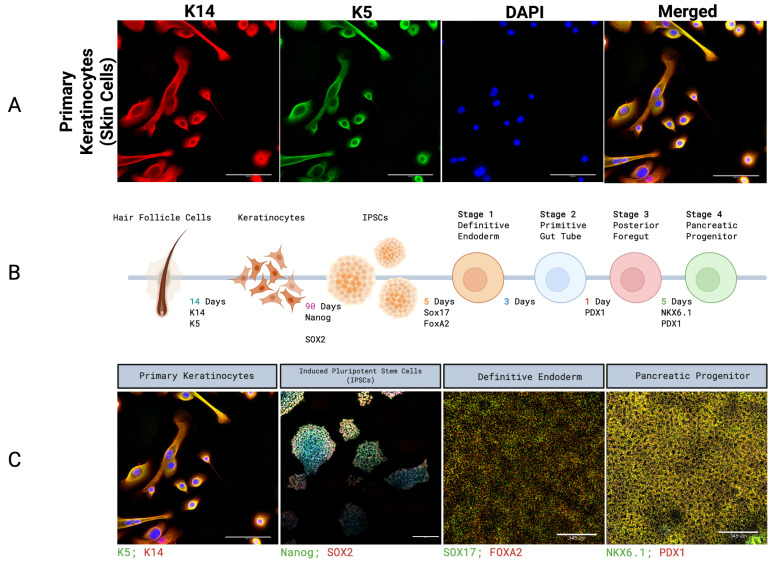
Characterization and reprogramming of HF-derived keratinocytes. (**A**) Immunofluorescence staining of primary keratinocytes showing expression of keratinocyte markers K5 (green) and K14 (red). Scale bars = 100 µm. (**B**) Schematic representation of the experimental workflow for reprogramming keratinocytes into iPSCs. (**C**) Representative immunofluorescence images of key differentiation stages. Scale bars = 100 µm (keratinocytes), 200 µm (iPSCs), and 345 µm (definitive endoderm and pancreatic progenitor).

Using a direct explant culture technique, keratinocytes selectively migrated out of the outer root sheath over the first two weeks of culture. Morphologically, cultured keratinocytes exhibited a characteristic cobblestone appearance, a hallmark of healthy epithelial colonies, and demonstrated robust colony formation and consistent proliferative potential in serial passages. In the first ten passages, we confirmed the stable expression of key basal epithelial markers, including cytokeratin 14 (K14) and cytokeratin 5 (K5), indicating the maintenance of a progenitor phenotype (Figure 1A).

### 2.2. iPSC Reprogramming and Pancreatic Differentiation

Keratinocytes isolated from human hair follicles were successfully reprogrammed into iPSCs and subsequently differentiated to definitive endoderm and pancreatic progenitor lineages (Figure 1B). This process recapitulates early developmental transitions and highlights the suitability of keratinocyte-derived iPSCs for endodermal differentiation. Primary keratinocytes expressed K5 and K14, characteristic of epithelial origin, whereas reprogrammed colonies displayed the pluripotency markers Nanog and SOX2. Following directed differentiation, cells expressed SOX17 and FOXA2, consistent with definitive endoderm identity, and further maturation yielded double-positive pancreatic progenitors of NKX6.1 and PDX1 (Figure 1C). Together, these findings demonstrate that keratinocyte-derived iPSCs retain the capacity to efficiently generate intermediates of the pancreatic lineage under appropriate differentiation signals.

### 2.3. MSC Isolation, Characterization and Differentiation

**Figure 2:**
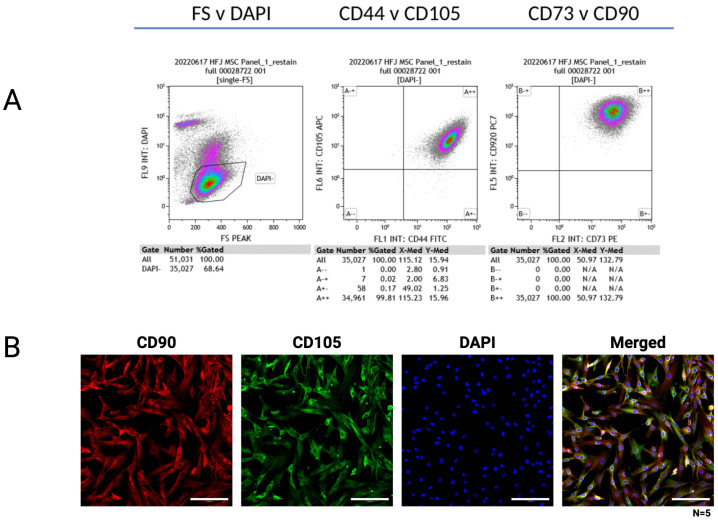
Characterization of human hair follicle-derived mesenchymal stem cells (HF-MSCs). (**A**) Flow cytometric analysis showing positive expression of MSC surface markers CD44, CD73, CD90, and CD105. (**B**) Immunofluorescence staining confirming high expression of CD90 (red) and CD105 (green), with nuclear counterstaining (DAPI, blue). Scale bars = 100 µm.

**Figure 3:**
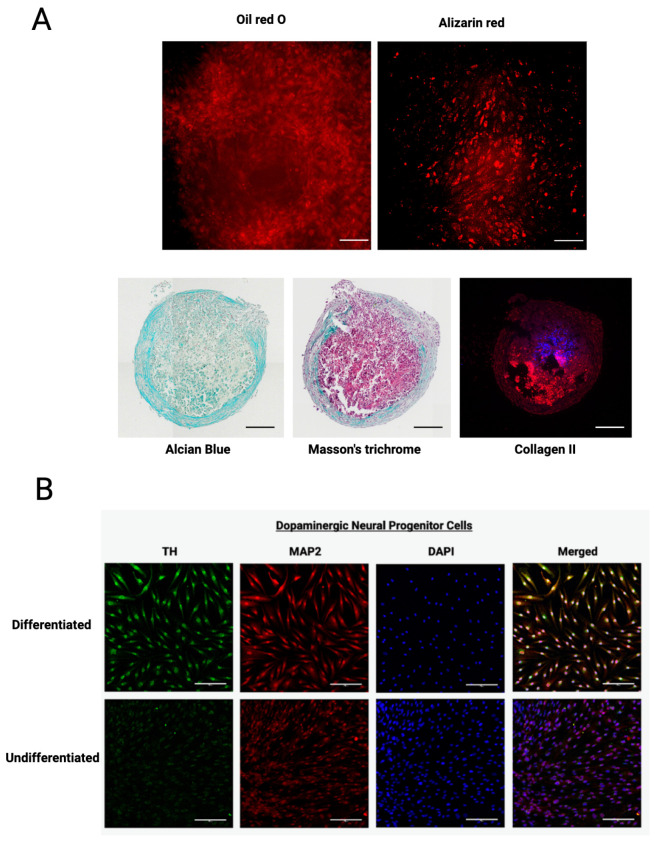
Differentiation potential of human hair follicle-derived mesenchymal stem cells (HF-MSCs). (**A**) Trilineage differentiation of HF-MSCs. Adipogenic, osteogenic, and chondrogenic differentiation confirmed via Oil Red O, Alizarin Red S, and chondrogenic-specific staining (Alcian Blue, Masson’s Trichrome, and Collagen II), respectively. Scale bars = 200 µm (top panels) and 100 µm (bottom panels). (**B**) Neural induction of HF-MSCs showing dopaminergic commitment and neuronal morphology with expression of tyrosine hydroxylase (TH) and microtubule-associated protein 2 (MAP2). Nuclei were counterstained with DAPI. Scale bars = 100 µm.

Following the isolation of MSCs from plucked anagen-phase hair follicles, adherent spindle-shaped cells were observed migrating from the dermal papilla region and were subsequently expanded. Flow cytometric profiling quantitatively confirmed that isolated HF-MSCs exhibited a typical mesenchymal immunophenotype, strongly expressing the canonical markers CD44, CD73, CD90, and CD105 (Figure 2A). A representative subset of these markers (CD90 and CD105) was further visualized via immunofluorescence to confirm spatial distribution and morphology in the adherent spindle-shaped cells (Figure 2B). Following phenotypic characterization, HF-MSCs were evaluated for their multilineage potential. Under lineage-specific induction, cells acquired adipogenic, osteogenic, and chondrogenic phenotypes, confirming classical mesodermal plasticity (Figure 3A). Furthermore, HF-MSCs exhibited neurogenic-like differentiation potential under inductive conditions, adopting elongated morphologies reminiscent of neurons and expressing the dopaminergic enzyme tyrosine hydroxylase (TH) alongside the neuronal cytoskeletal marker microtubule-associated protein 2 (MAP2) (Figure 3B).

### 2.4. Characterization and Regenerative Potential of the HF Secretome

**Figure 4:**
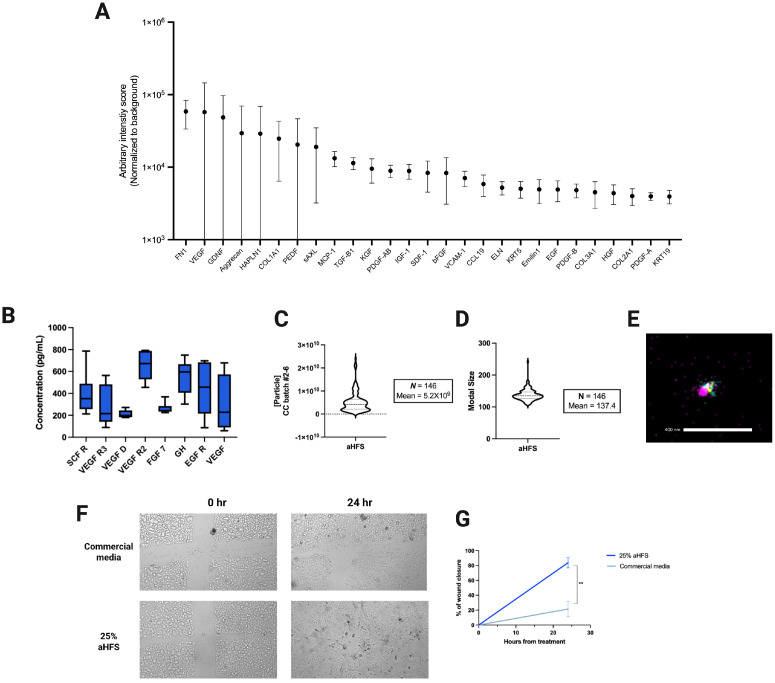
Characterization of the autologous hair follicle secretome (aHFS). (**A**) Semi-quantitative analysis of selected protein analytes in aHFS using a Quantibody^®^ glass slide-based array (RayBiotech). Data represent arbitrary intensity values normalized to background (*n* = 3). (**B**) Concentrations of selected growth factors and cytokines in aHFS (*n* = 6). Nanoparticle tracking analysis (NTA) showing particle concentration (**C**) and modal size distribution (**D**) in aHFS samples (*n* = 146). (**E**) ONI super-resolution image of exosomes present in aHFS lyophilized secretome. Exosomes were triple-labeled for tetraspanin markers CD81 (purple), CD63 (yellow), and CD9 (teal). (**F**) Representative brightfield images from a scratch wound assay using primary keratinocytes treated with commercial media or 25% aHFS, imaged at 0 and 24 h. (**G**) Quantification of wound closure over time. Data are shown as mean ± SEM. ** p<0.01 vs. control.

The biological complexity of the hair follicle secretome (aHFS) was further evaluated using a semi-quantitative protein array to characterize the secretome derived from hair follicle mesenchymal stem cells isolated from three healthy adult donors. Signal intensities were normalized to background media. Detected proteins included VEGF-A, fibronectin (FN1), and Aggrecan, among others associated with tissue regeneration and immune modulation, consistent with previously published proteomic analyses of the hair follicle environment (Figure 4A). Further characterization of aHFS prepared using hair follicles from six healthy donors revealed concentrations in the picogram per ml range for canonical regenerative cytokines such as VEGF-A, GH, and FGF-7 (Figure 4B). Nanoparticle tracking analysis confirmed the secretome’s vesicular content, with modal particle sizes centered around 137 nm, consistent with the size of exosomes. The average particle count among samples from 146 participants was measured as $5.9\times 10^{9}$ particles per ml (Figure 4C,D). Super-resolution imaging provided additional confirmation of vesicle identity, with triple-labeling for the tetraspanins CD81, CD63, and CD9 verifying the presence of exosomes within the lyophilized preparation (Figure 4E).

The wound healing potential of aHFS was also tested in an in vitro scratch assay using human keratinocytes. Cells treated with 25% aHFS in basal medium exhibited significantly better wound closure at 24 h compared to those treated with serum-free keratinocyte media (p<0.005, unpaired Student’s *t*-test). Phase-contrast imaging confirmed greater cellular migration and monolayer restoration in the aHFS-treated group (Figure 4F,G). These findings support the capacity of the HF-derived secretome to accelerate epithelial repair through paracrine signaling.

## 3. Discussion

The present study underscores the multidimensional regenerative capacity of the human hair follicle, which acts as a highly accessible reservoir for both somatic and mesenchymal stem cells. A persistent challenge in the clinical translation of autologous cell therapies remains the reliance on invasive tissue harvesting, which is frequently coupled with harsh enzymatic dissociation protocols [29]. These conventional methods can induce cellular stress and alter baseline cellular phenotypes [23]. In contrast, the present study establishes a streamlined, enzyme-free explant methodology utilizing non-invasively plucked hair follicles. We demonstrate that this approach successfully isolates and expands both HF-derived keratinocytes and MSCs while preserving their native progenitor characteristics, thus mitigating culture-induced artifacts.

Importantly, our explant methodology yielded a robust population of outer root sheath keratinocytes, which serve as a highly efficient source for cellular reprogramming. As established in the literature, keratinocytes exhibit superior reprogramming efficiency compared to dermal fibroblasts, a distinct advantage driven by their endogenous expression of key epithelial transcription factors [29,30]. Leveraging this baseline advantage, we successfully reprogrammed these HF-derived keratinocytes into iPSCs using an integration-free episomal approach. We subsequently demonstrated their capacity to differentiate across germ layers into definitive endoderm and pancreatic progenitor lineages (PDX1+/NKX6.1+). While the comprehensive functional characterization of these keratinocyte-derived pancreatic progenitors has been extensively detailed in our previous work [12], their generation here confirms that non-invasively plucked hair follicles reliably provide high-quality keratinocytes for potential use in regenerative therapies.

In parallel, the mesenchymal stem cells (HF-MSCs) isolated via our non-invasive explant method exhibited profound developmental versatility. Beyond classical mesodermal differentiation, HF-MSCs demonstrated the capacity for neurogenic-like induction, adopting dopaminergic phenotypes (TH+/MAP2+). This intrinsic multilineage capacity, achieved without genetic manipulation, underscores the plucked follicle as a highly competitive alternative to traditional sources of bone marrow or stem cells derived from adipose tissue. Furthermore, recent advances in expanding HF-MSCs within fully defined serum-free media enhance their clinical translatability for tissue engineering and immune modulation [31].

Beyond direct cellular integration, the therapeutic efficacy of HF-derived cells is highly mediated by their potent paracrine activity [17,18]. Our characterization of the autologous hair follicle secretome (aHFS) revealed a robust concentration of regenerative cytokines, growth factors, and extracellular vesicles. A major innovation of this work lies in the scale and rigor of our cell-free analysis. While previous reports have described the regenerative properties of HF-derived conditioned media [32] and its associated extracellular vesicles [33], this study provides a uniquely comprehensive functional and vesicular characterization of the autologous hair follicle secretome (aHFS). Importantly, the secretome was generated within a chemically defined, serum-free environment. This methodological rigor ensures that the potent wound-closure capabilities and specific cytokine enrichments (e.g., VEGF-A, FGF-7) observed in our assays are driven exclusively by autologous paracrine factors and extracellular vesicles, free from the confounding influence of exogenous animal proteins. The pronounced wound-closure capabilities demonstrated in our in vitro keratinocyte scratch assays firmly confirm this regenerative capacity.

Cellular aging remains a fundamental barrier to scalable cell-based therapies. Extended in vitro expansion and chronological aging drive variability in gene expression, upregulate senescence markers, and ultimately diminish differentiation potential [23]. Although iPSC reprogramming can partially reset age-associated molecular phenotypes, it remains insufficient to completely erase accumulated DNA damage or epigenetic scarring [27,34]. Consequently, the functional utility of autologous cellular therapies is most robust when sourced from a biologically young tissue resource.

To address this biological limitation, the cryopreservation of intact, plucked hair follicles offers a highly practical strategy for personalized regenerative medicine. By circumventing the need for invasive biopsies, early-stage biobanking of these follicles arrests cellular aging at the point of collection, preserving native telomere length and mitochondrial integrity [28]. This approach secures a single, non-invasively acquired tissue source that can simultaneously yield scalable populations for patient-specific therapies, offering an accessible, multi-modal framework for the future of autologous interventions.

Despite the robust regenerative potential demonstrated, this study has two primary limitations. First, while our profiling identified key regenerative cytokines and confirmed the presence of exosomes, the autologous secretome is an inherently complex conditioned medium. This biochemical complexity, combined with the natural biological variability observed across human donors, makes it challenging to pinpoint the exact contributions of specific soluble versus vesicular fractions. Second, although our initial protein analysis provides valuable foundational data, the cohort size for this specific profiling was limited. More detailed demographic stratification in larger studies is required to comprehensively understand how variables such as donor sex, genetics, and underlying health conditions might influence individual secretome profiles.

## 4. Materials and Methods

### 4.1. Hair Follicle Collection

Hair follicles were collected from the occipital region of the scalp of 28 participants. Participants consented to the collection and subsequent use of their follicles. The follicles were collected using a plucking motion and transported in Acorn’s Cell Transportation Medium^TM^ (Gibco, Emeryville, CA, USA) for further analysis.

### 4.2. Primary Cell Culture Using the Explant Culture Method

The samples were transferred into 35 mm sterile Petri dishes (Thermo Fisher Scientific, Waltham, MA, USA; Cat. #153066) containing CTS-DPBS without Mg^2+^ and Ca^2+^ (Gibco, Grand Island, NY, USA; Cat. #A1285601) and then washed for 3 min in the solution. The dishes were transferred into the ISO-7 cleanroom for further processing. For each participant, 10 follicles were trimmed and placed in the middle of a 48-well tissue culture plate (Nunc, Roskilde, Denmark; Cat. #150687) coated with Matrigel (Corning, New York, NY, USA; Cat. #354230). Only follicles containing an intact ORS were selected. To mitigate against the follicles drying, a drop of KSR medium—comprising 20% Knockout Serum Replacement (Gibco, Grand Island, NY, USA; Cat. #10828028) in DMEM/F12 (Gibco; Cat. #11320033), supplemented with 1× MEM Non-Essential Amino Acids (Gibco; Cat. #11140050), 0.1 mM β-Mercaptoethanol (Gibco; Cat. #21985023), and 1× Antibiotic-Antimycotic (Gibco; Cat. #15240062)—was added to each well prior to plating. Once all the follicles were plated, the media were removed, and a fresh drop of media (50 μL) containing 10 ng/μL bFGF (Thermo Fisher Scientific, Waltham, MA, USA; Cat. #PHG0024) was added on top of each follicle. The plates were placed in a 37 °C incubator at 5% CO_2_ overnight. The next morning, each well was flooded with 150 μL of media. A complete media change was conducted every other day.

### 4.3. Keratinocyte Isolation and Expansion

At 75% confluency, cells were enzymatically dissociated using 200 μL of TrypLE Express (Gibco, Grand Island, NY, USA; Cat. #12604013) per well. The suspension of cells was pooled for each participant and centrifuged at 300× *g* for 5 min. The resulting supernatant was discarded, and the pellets were resuspended in 1 mL of DermaCult Keratinocyte Expansion Medium (STEMCELL Technologies, Vancouver, BC, Canada; Cat. #05420) supplemented with Hydrocortisone (STEMCELL Technologies; Cat. #07925). The solution was added to a 12-well tissue culture plate (Greiner Bio-One, Frickenhausen, Germany; Cat. #665180) coated with Rat Tail Collagen Type I (Gibco, Grand Island, NY, USA; Cat. #A1048301). The cells were incubated in a 37 °C incubator at 5% CO_2_, and the media were changed every other day. The cells were expanded until 80% confluency and then passaged onto 6-well tissue culture plates (Greiner Bio-One; Cat. #657160) at a density of $3\times 10^{4}$/cm^2^. At each passage, cells were counted using a hemocytometer, and keratinocyte expansion rates were extrapolated. On passage 3, the cells were taken for reprogramming.

### 4.4. iPSC Reprogramming and Directed Differentiation

Reprogramming of HF-derived keratinocytes into induced pluripotent stem cells (iPSCs) was performed using an integration-free episomal approach, as previously described. The generated iPSCs were maintained and expanded in Essential 8 medium (Thermo Fisher Scientific, Waltham, MA, USA; Cat. #A2656101). Successful reprogramming was confirmed prior to differentiation via the expression of core pluripotency markers Nanog and SOX2. To initiate directed differentiation, iPSCs were dissociated and seeded onto 24-well plates coated with Geltrex (Thermo Fisher Scientific, Waltham, MA, USA; Cat. #A1413301) at lineage-appropriate densities. Stage-specific differentiation was driven using the STEMdiff Trilineage Differentiation Kit (STEMCELL Technologies, Vancouver, BC, Canada; Cat. #05230) according to the manufacturer’s guidelines, successfully inducing the sequential expression of definitive endoderm markers (SOX17, FOXA2) and subsequent pancreatic progenitor markers (NKX6.1, PDX1).

### 4.5. MSC Isolation and Characterization

Mesenchymal stem cells (MSCs) were isolated by plating plucked hair follicles directly onto culture plates. Prior to plating, the anagen phase of the follicles was confirmed visually using a Leica DMi1 inverted phase-contrast microscope (Leica Microsystems, Wetzlar, Germany). Adherent, spindle-shaped cells were allowed to migrate from the dermal papilla region. These cells were subsequently harvested and expanded in DMEM/F12 basal medium (Thermo Fisher Scientific, Waltham, MA, USA; Cat. #11320033) supplemented with 10% Fetal Bovine Serum (FBS; Thermo Fisher Scientific; Cat. #A5256801) under standard incubation conditions (37 °C, 5% CO_2_). Cells were utilized between passages 2 and 5.

### 4.6. Flow Cytometry

HF-MSCs were harvested using TrypLE Express (Gibco, Grand Island, NY, USA; Cat. #12604013) and centrifuged (300× *g*, 5 min). Pellets were resuspended in 2% FBS/PBS and blocked with Human TruStain FcX (BioLegend, San Diego, CA, USA; Cat. #422301) for 10 min at room temperature. Cells were incubated with fluorophore-conjugated antibodies against CD44 (Invitrogen, Thermo Fisher Scientific, Waltham, MA, USA; Cat. #11-0441-82), CD73 (BioLegend, San Diego, CA, USA; Cat. #344004), CD90 (Invitrogen; Cat. #25-0909-41), and CD105 (BioLegend; Cat. #323208) for 20 min on ice in the dark. After washing twice, cells were stained with DAPI (Thermo Fisher Scientific, Waltham, MA, USA; Cat. #D1306) for 5 min. Data were acquired on a BD FACSCelesta flow cytometer (BD Biosciences, San Jose, CA, USA) and analyzed using FlowJo software (v10, BD Biosciences).

### 4.7. Multilineage Differentiation

To evaluate multilineage mesodermal plasticity, HF-MSCs underwent lineage-specific induction. Cells were cultured in respective adipogenic (MesenCult^TM^ Adipogenic Differentiation Kit, STEMCELL Technologies, Vancouver, BC, Canada; Cat. #05412), osteogenic (MesenCult^TM^ Osteogenic Differentiation Kit, STEMCELL Technologies; Cat. #05465), and chondrogenic (MesenCult^TM^-ACF Chondrogenic Differentiation Kit, STEMCELL Technologies; Cat. #05455) induction media. Differentiation was confirmed using Oil Red O (Thermo Fisher Scientific, Waltham, MA, USA; Cat. #189400250), Alizarin Red S (Thermo Fisher Scientific; Cat. #AA42746AE), and Alcian Blue staining (performed at The Centre for Phenogenomics Pathology Core, Hospital for Sick Children, Toronto, ON, Canada). To achieve the high contrast required for detecting faint micro-lipid droplets and early-stage mineral deposits, the intrinsic fluorescent properties of Oil Red O and Alizarin Red S were utilized for imaging. Chondrogenesis was further confirmed using Masson’s Trichrome and Collagen II staining (The Centre for Phenogenomics, Toronto, ON, Canada).

Neurogenic-like differentiation was induced under specific conditions as previously described [35]. Briefly, HF-MSCs (passages 3–5) at 70–80% confluency were dissociated using TrypLE Express (Gibco, Grand Island, NY, USA; Cat. #12604013) and seeded into 12-well plates in standard 10% FBS media in DMEM/F12 (Gibco; Cat. #11320033). Following overnight adhesion at 37 °C, the growth medium was replaced with a neural induction medium consisting of Neurobasal Medium (Gibco; Cat. #21103049) supplemented with B-27 (0.25×, Thermo Fisher Scientific, Waltham, MA, USA; Cat. #17504044), 250 ng/mL SHH (Thermo Fisher Scientific; Cat. #PHC7014), 100 ng/mL FGF8 (Thermo Fisher Scientific; Cat. #PHG0271), and 50 ng/mL bFGF (Thermo Fisher Scientific; Cat. #13256029). The cells were incubated at 37 °C without media changes for 9 days. On day 9, the culture was supplemented with 50 ng/mL BDNF (Thermo Fisher Scientific; Cat. #RP-8642) and incubated for an additional 3 days (12 days total). To characterize the neurogenic potential, cells were fixed in 4% PFA and stained with primary antibodies against Tyrosine Hydroxylase (TH; Abcam, Cambridge, UK; Cat. #ab112) and MAP2 (Abcam; Cat. #ab32454) prior to visualization.

### 4.8. Immunofluorescence

For immunofluorescence analysis, cells were seeded into 12-well cell culture plates (Nunc, Roskilde, Denmark; Cat. #150628) and fixed in 3.7% paraformaldehyde (Thermo Fisher Scientific, Waltham, MA, USA; Cat. #A11313.22) for 20 min at room temperature. Following three washes with PBS, cells were permeabilized using 0.1% Triton X-100 (Thermo Fisher Scientific; Cat. #A16046.AE) for 10 min and subsequently blocked with 1% BSA (Thermo Fisher Scientific; Cat. #37525) for 1 h at room temperature. Cells were then incubated overnight at 4 °C with primary antibodies targeting specific lineage markers: K5 (Invitrogen, Thermo Fisher Scientific, Waltham, MA, USA; Cat. #PA5-32465) and K14 (Invitrogen; Cat. #MA5-11599) for keratinocyte maintenance; CD90 (Invitrogen; Cat. #MA1-35307) and CD105 (Invitrogen; Cat. #MA5-11854) for MSCs; Nanog (Invitrogen; Cat. #PA1-097), SOX2 (Invitrogen; Cat. #MA1-014), SOX17 (Invitrogen; Cat. #703465), FOXA2 (Invitrogen; Cat. #MA5-31362), NKX6.1 (Invitrogen; Cat. #PA5-106121), and PDX1 (Invitrogen; Cat. #PA5-85093) for differentiation; and TH (Invitrogen; Cat. #OPA1-04050) and MAP2 (Invitrogen; Cat. #MA5-12826). After 24 h, cells were incubated with species-specific secondary antibodies for 1 h. Nuclei were counterstained with DAPI (Thermo Fisher Scientific; Cat. #D1306) for 5 min. Plates were imaged using the IXM Confocal System (Molecular Devices, San Jose, CA, USA).

### 4.9. Secretome Collection and Protein Arrays

Ten anagen hair follicles (confirmed visually as described in Section 4.5) for each participant were trimmed and plated in duplicate in 48-well plates. Cells were cultured in a serum-free, xeno-free, chemically defined medium (DMEM/F12 supplemented with a proprietary blend of factors; Acorn Biolabs, Toronto, ON, Canada). After 48 h of cell culture, the secretome was collected. Throughout the culture period, cells were evaluated daily via phase-contrast microscopy to confirm monolayer integrity and the absence of cellular detachment. The conditioned medium was centrifuged at 300× *g* for 5 min to remove cellular debris, filtered using 0.2 μm filters (Amicon, Merck Millipore, Burlington, MA, USA; Cat. #UFC500324), and then lyophilized. A semi-quantitative Quantibody^®^ glass slide-based cytokine array (RayBiotech, Peachtree Corners, GA, USA; Cat. #QAM-CAA-4000) was utilized to profile growth factors and cytokines. Signal intensities were normalized to background media. Specific analyte concentrations (VEGF-A, GH, FGF-7) were further quantified via ELISA assays (RayBiotech; Cat. #ELH-VEGF-1).

### 4.10. EV Characterization

The vesicular fraction was obtained from the aHFS using the Total Exosome Isolation Kit (from cell culture media) (Invitrogen, Thermo Fisher Scientific, Waltham, MA, USA; Cat. #44783598). The vesicular content was characterized using Nanoparticle Tracking Analysis (NTA) on a NanoSight NS300 instrument (Malvern Panalytical, Malvern, UK) to determine modal particle size and concentration (n=146). Super-resolution microscopy was performed using the ONI platform (ONI, Oxford, UK). Lyophilized secretome preparations were triple-labeled for the canonical exosome tetraspanins using antibodies against CD81 (Invitrogen; Cat. #MA5-13548), CD63 (Invitrogen; Cat. #MA5-14717), and CD9 (Invitrogen; Cat. #MA5-14712).

### 4.11. Scratch Assay

An in vitro scratch wound assay was performed using primary human keratinocytes to evaluate directed cellular migration. Cells were seeded in 12-well plates (Greiner Bio-One, Frickenhausen, Germany; Cat. #665180) and grown to 90% confluence in KGM (Keratinocyte Growth Medium) (Lonza, Basel, Switzerland; Cat. #CC-3111) to form a uniform monolayer. A standardized linear scratch was created in the center of each well using a sterile 200 μL pipette tip held at a constant angle and pressure to ensure a uniform wound width. To guarantee that the exact same field of view was analyzed over time, reference lines were drawn on the underside of the plate, and images were captured at the intersection of the scratch and the reference marker. Following scratching, cells were gently washed with PBS to remove detached debris and treated with 25% aHFS diluted in basal medium. KGM-Gold CaFree BulletKit (Lonza, Basel, Switzerland; Cat. #00195130) served as the standard control, ensuring that baseline measurements were not confounded by undefined serum proteins. The wound closure was monitored, and phase-contrast images were captured at 0 and 24 h using a Leica DMi1 microscope (Leica Microsystems, Wetzlar, Germany) to quantify percentage area closure.

### 4.12. Statistical Analysis

Quantitative data are presented as the mean ± standard error of the mean (SEM) from at least three independent experiments (n≥3). Statistical significance between two groups was determined using an unpaired Student’s *t*-test. For comparisons involving more than two groups, a one-way analysis of variance (ANOVA) followed by Tukey’s post hoc test for multiple comparisons was performed. All statistical analyses were conducted using GraphPad Prism software (v8.0; GraphPad Software, San Diego, CA, USA). A *p*-value <0.05 was considered statistically significant (* p<0.05, ** p<0.01).

## 5. Conclusions

The human hair follicle is a clinically accessible, biologically diverse tissue with growing relevance in personalized regenerative medicine. It contains multiple somatic and stem cell populations capable of contributing to epithelial, mesenchymal, and neural regeneration. In addition, the hair follicle-derived secretome provides a rich source of trophic and immunomodulatory factors that support tissue repair through paracrine mechanisms.

Given the expanding clinical pipeline of autologous cell-based therapies, establishing robust and accessible sources of regenerative cells is a critical challenge. Cryopreservation of intact follicles at early life stages offers a practical strategy to preserve cellular function and molecular integrity, minimizing the effects of aging and culture-induced drift. This biobanking approach enables flexible autologous applications, including iPSC generation, secretome therapies, and bioengineering. Upon the collection of young cells and storage under optimal cryogenic conditions, these follicles become a biologically stable source of regenerative material positioned to support a future landscape of personalized medicine.

## Data Availability

All data are contained within the manuscript.
